# Combining 3D human in vitro methods for a 3Rs evaluation of novel titanium surfaces in orthopaedic applications

**DOI:** 10.1002/bit.25919

**Published:** 2016-01-21

**Authors:** G. Stevenson, S. Rehman, E. Draper, E. Hernández‐Nava, J. Hunt, J.W. Haycock

**Affiliations:** ^1^ JRI Orthopaedics Ltd.SheffieldUnited Kingdom; ^2^ Mercury CentreUniversity of SheffieldSheffieldUnited Kingdom; ^3^ Department of Materials Science and EngineeringUniversity of SheffieldSheffieldS3 7HQUnited Kingdom

**Keywords:** additive manufacturing, in vitro test, osteoblast, plasma spraying, surface topography, titanium alloy

## Abstract

In this study, we report on a group of complementary human osteoblast in vitro test methods for the preclinical evaluation of 3D porous titanium surfaces. The surfaces were prepared by additive manufacturing (electron beam melting [EBM]) and plasma spraying, allowing the creation of complex lattice surface geometries. Physical properties of the surfaces were characterized by SEM and profilometry and 3D in vitro cell culture using human osteoblasts. Primary human osteoblast cells were found to elicit greater differences between titanium sample surfaces than an MG63 osteoblast‐like cell line, particularly in terms of cell survival. Surface morphology was associated with higher osteoblast metabolic activity and mineralization on rougher titanium plasma spray coated surfaces than smoother surfaces. Differences in osteoblast survival and metabolic activity on titanium lattice structures were also found, despite analogous surface morphology at the cellular level. 3D confocal microscopy identified osteoblast organization within complex titanium surface geometries, adhesion, spreading, and alignment to the biomaterial strut geometries. Mineralized nodule formation throughout the lattice structures was also observed, and indicative of early markers of bone in‐growth on such materials. Testing methods such as those presented are not traditionally considered by medical device manufacturers, but we suggest have value as an increasingly vital tool in efficiently translating pre‐clinical studies, especially in balance with current regulatory practice, commercial demands, the 3Rs, and the relative merits of in vitro and in vivo studies. Biotechnol. Bioeng. 2016;113: 1586–1599. © 2015 The Authors. *Biotechnology and Bioengineering* Published by Wiley Periodicals, Inc.

## Introduction

Hip replacements are one of the most common orthopaedic procedures with over 90,000 operations performed in 2013 in England and Wales alone (NJR, 2014), with the number of procedures set to rise due to an aging population. Coupled with this, there is an increasing need for the treatment of younger and more active patients, which places greater demands on the implants used. Long‐term implant success in these younger and more active patients depends greatly on effective biological fixation by bone in growth (Kienapfel et al., 1999; McLaughlin and Lee, 2011). In order to achieve this, surgeons use cementless press‐fit prostheses with surface modifications to promote bone in‐growth as well as initial primary fixation by mechanical interlocking (Sammons, 2011). Traditionally, modification of metallic implant surfaces has been achieved using a variety of techniques including grit blasting with aluminium oxide, plasma spraying with titanium, and/or hydroxyapatite, sintering metal beads onto the implant surface, and diffusion bonding of fibre metal mesh (Levine, 2008; Sammons, 2011). Porous surfaces have been shown to have a superior bony response than surfaces treated by grit‐blasting alone, highlighting that surface texture is important in achieving good biological fixation (Dávid et al., 1995).

Potential problems with the use of coatings to create surface roughness and porosity include coating delamination and cracking under fatigue (Murr et al., 2012), as well as a limit to the volume of porosity achieved by these methods (Bobyn et al., 1999). To overcome such issues, additive manufacture (AM) has become an area of growing interest for manufacturing parts with complex surface geometries. AM offers design freedoms, which enable the production of geometries unattainable by traditional machining methods. AM techniques such as electron beam melting (EBM) can create parts of high complexity, achieved by sequentially melting layers of metal powder to the geometry of a computer‐aided design model of the desired component (Heinl et al., 2007; Parthasarathy et al., 2010; Thomsen et al., 2009). Components can therefore be produced with a porous surface as an integral part of the implant, rather than as an additional coating. The increased amount of porosity achieved by AM has been shown to improve implant fixation strength in vivo, in both sheep and goat models (Biemond et al., 2011; Stübinger et al., 2013).

In order to meet medical device regulations, the testing and characterization of new surfaces is essential, not only to fulfill basic requirements, but to go “Beyond Compliance” and ensure that every stage of device development is understood in detail (Northgate, 2014). In vitro preclinical evaluation is particularly valuable for determining whether a material is suitable for in vivo use and predicting the in vivo response. Traditionally, implant materials are studied directly in animal models, with minimal understanding of the expected biological response. Having an understanding of the response to materials and surfaces at a cellular level means that in vivo experiments can be more targeted.

The development of more complex in vitro methods addresses the Replacement, Reduction, and Refinement (3Rs) framework for humane animal research, which is now becoming embedded in national and international legislation regulating the use of animals in scientific procedures (NC3Rs, 2014; Russell and Burch, 1959). Against this, the 3Rs framework must, however, maintain high quality science when developing alternative approaches that seek to avoid the use of animals. There is therefore a continual need for better experimental models and methods when predicting the efficacy and safety of new implants or medicines. Human cell culture techniques commonly use MG63 osteoblast cells; however, the use of normal human osteoblasts is arguably more relevant, and enables the study of several important functions including adhesion, metabolic activity, viability, phenotype, and differentiation marker analysis. The use of 3D confocal laser scanning microscopy (CLSM) in vitro allows assessment of more complex surface geometries, with good spatial and axial resolution (Georgakoudi et al., 2008) of relevance to biomaterials with irregular surface structures such as porous substrates (Hearnden et al., 2011).

The present work reports on the development of a 3D in vitro approach using human osteoblasts, together with a range of readily applicable techniques to assess the physical and biological properties of surfaces for potential orthopaedic applications. Titanium surfaces of differing roughness, prepared using traditional vacuum plasma spraying and porous titanium substrates prepared using EBM were assessed. Cellular attachment and proliferation of a bone‐like osteosarcoma cell line (MG63) were used initially with data confirmed and extended to the use of primary human adult osteoblast cells, where differentiation and mineralization were studied to investigate the potential for bone formation on the surfaces. Confocal microscopy z‐depths of up to 1,875 μm were examined for irregular surface topographies allowing interpretation of osteoblast adhesion, viability, and phenotypic organization.

## Methods

### Fabrication of Vacuum Plasma Sprayed Titanium Substrates

Discs of Ti6Al4V alloy (12 mm diameter × 9 mm height) were grit blasted with Al_2_O_3_ blast media to roughen the surface. Roughened discs were cleaned by ultrasonication in deionized water then dried in air before a coating of commercially pure titanium (cpTi) was applied by vacuum plasma spraying (VPS) to a thickness of approximately 50 μm. Two cpTi powder sizes, −100 and −325 mesh, were used to obtain coarse and fine titanium coatings, respectively. These coatings are henceforth termed Ti Coarse and Ti Fine.

### Fabrication of Porous Lattice Substrates

**Figure 1:**
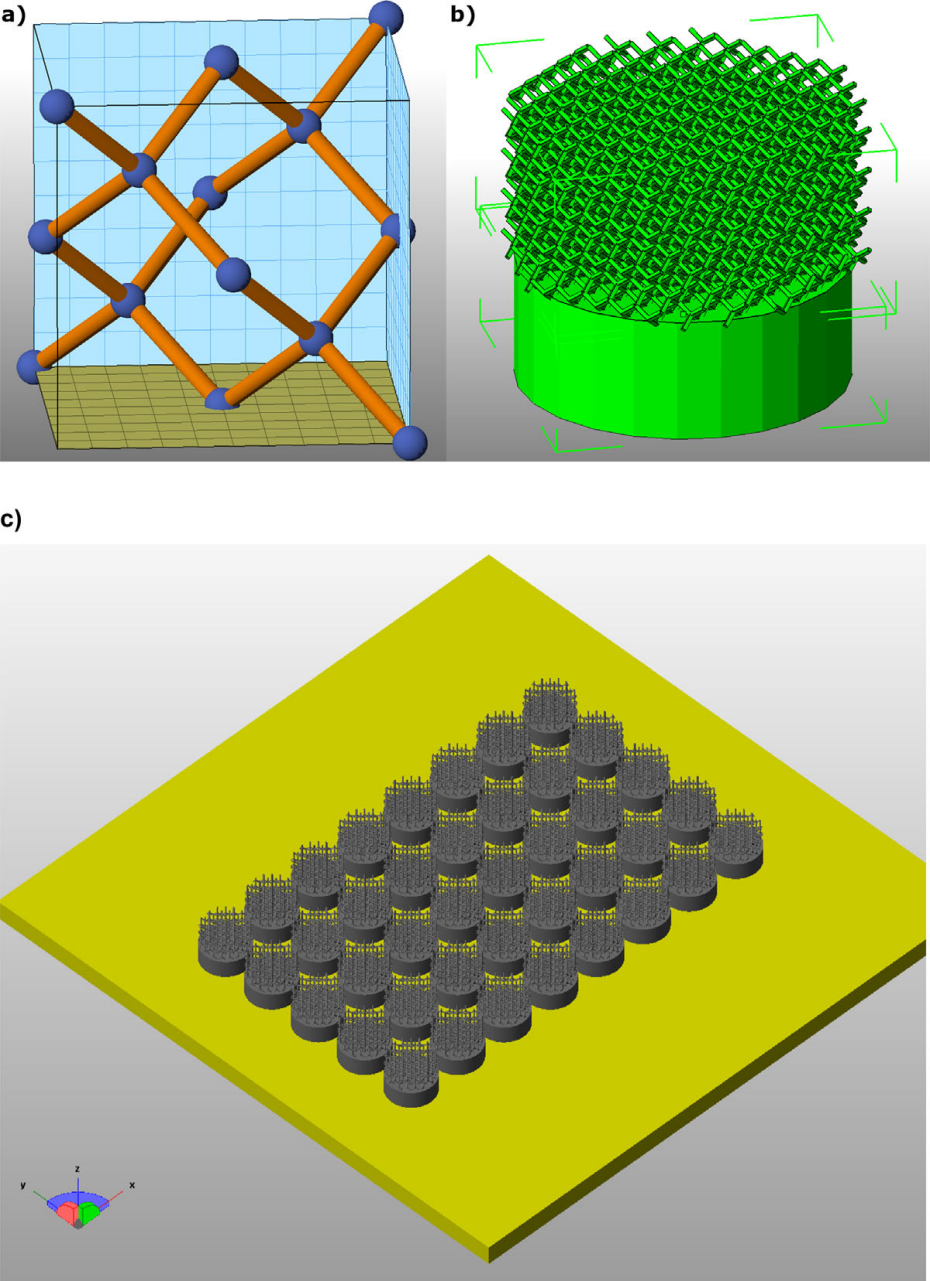
(**a**) Design of the diamond unit cell, with spheres schematically highlighting the connectivity points only. (**b**) The solid and porous part on the final model with 1.5 mm^3^ unit cell size. (**c**) Build layout of multiple samples with direction of build in the Z‐axis.

Lattice structures were fabricated using EBM (Eitel, 2013; Van Noort, 2012). The sample design of a cylinder (12 mm diameter and 11 mm height) comprised a half solid and half lattice sketched using Netfabb® (Netfabb GmbH, Lupburg, Germany) prototyping software. A diamond cubic unit cell was designed (Fig. 1a) and reproduced in the constraint volume of the lattice half of the sample. All samples were designed with a truss diameter of 400 μm, with the unit cell size (Fig. 1b) set to 1.5 mm^3^ for the first batch and 2.5 mm^3^ for the second batch, and replicated 40 times to produce the required number of samples (Fig. 1c). Build files were sliced into 70 μm layers and exported as “SLC” files (contour representation) compatible with an Arcam S12 EBM machine. Titanium alloy grade 23 was used (an extra low interstitial [ELI] version of Ti6Al4V alloy), used in biomedical applications. The gas atomized powder was 45–105 μm. The layer of powder was preheated to provide a partially sintered bed, the outline or contour of the shape was melted and the material inside the contours was melted with a hatch pattern. Two melting strategies were employed: (i) for the solid part, a higher energy input with a fast defocused electron beam was used and (ii) for the lattice section a slow beam with a sharp focus utilized. An offset to contour was employed, as is common practice for small trusses, to prevent thin sections from being oversized in the x‐y plane. Full details of beam parameters are given in Table I. Un‐melted powder was removed by blasting with a compressed air line. All samples were cleaned in deionized water, air dried, and vacuum packed. Samples were sterilized by gamma irradiation at a dose of 25 kGy (Swann Morton Ltd., Sheffield, U.K.).

**Table I:** (a) Beam parameters used during the manufacture of EBM samples. (b) Summary of the tests performed in this study with time points for cell culture studies

(a)	Beam current (mA)	Beam speed (mm/s)	Focused beam	Offset to contour
Solid section	14–15	500	No	No
Lattice section	1.7–3.0	200	Yes	Yes

(b)	Characterization		Biological assessment—MG63 cell line & HOb cells			
Parameter	Surface morphology		Live/dead	Cell Viability	Cell morphology	Mineralization (HOb only)
Technique	SEM	Surface Roughness	SYTO9 & propidium iodide staining	MTT assay	Phalloidin‐TRITC & DAPI staining	Xylenol orange staining
Sample Type						
Fine titanium (Ti Fine)	√	√	24 h	24 h	24 h	14 day
			96 h	96 h	96 h	
				14 day		
Coarse titanium (Ti Coarse)	√	√	24 h	24 h	24 h	14 day
			96 h	96 h	96 h	
				14 day		
1.5 mm^3^ lattice	√	√	24 h	24 h	24 h	14 day
			96 h	96 h	96 h	
				14 day		
2.5 mm^3^ lattice	√	√	24 h	24 h	24 h	14 day
			96 h	96 h	96 h	
				14 day		

### Characterization of Surfaces Using Scanning Electron Microscopy (SEM)

SEM imaging was conducted on a JEOL JSM 6400 scanning electron microscope using an accelerating voltage of 10 kV with images recorded using I‐Scan image acquisition system.

### Surface Roughness Measurements

Surface roughness of the samples was measured using a Veeco DektaK 150 surface contact profilometer, with a stylus radius of 12.5 μm and a 3 mg load. Measurements were made with a scan length of 2,000 μm and duration of 60 s. For the EBM samples the surface roughness measurement was made on the solid base of the structure, as positioning of the stylus on to individual trusses was not possible.

### Culture of MG63 Human Osteosarcoma Cells

MG63 human osteosarcoma cells (ATCC, CRL‐1427, at passage 86) were cultured in Dulbecco's modified Eagle medium (DMEM) supplemented with 10% (v/v) foetal calf serum, 1% (w/v) glutamine, 1% (w/v) penicillin/streptomycin, and 0.25% (w/v) amphotericin B in a humidified atmosphere with 5% CO_2_ at 37°C. Cells were sub‐cultured every 4–5 days when at 80–90% confluency. Cell studies were carried out in 24‐well tissue‐culture plates (BD Biosciences, Oxford, U.K.) with each sample immersed in 1.5–2 mL cell growth medium. Cells were seeded at a density of 3,125 cells/cm^2^ and the culture medium was changed every 2–3 days. Cells at passage number 89–96 were used for experiments.

### Culture of Primary Human Osteoblast Cells

Primary human adult osteoblast cells (ECACC, Primary human osteoblast 406‐05a) were cultured in Dulbecco's modified Eagle medium (DMEM) supplemented with 10% (v/v) foetal calf serum, 1% (v/v) glutamine, 1% (v/v) penicillin/streptomycin, 0.25% (v/v) amphotericin B, and 0.05 % (w/v) L‐ascorbic acid in a humidified atmosphere with 5% CO_2_ at 37°C. Cells were sub‐cultured every 5–7 days when at 80% confluency. Cell studies were conducted in 24‐well tissue‐culture plates (BD Biosciences, Oxford, U.K.) with each sample immersed in 1.5–2 mL medium, seeded at a density of 25,000 cells/cm^2^. Culture medium was changed every 2–3 days and cells used at passages 3–7 for experiments.

### Determination of Cell Metabolic Activity

Cell metabolic activity was estimated using MTT assay to give an indirect measure of osteoblast cell number, and over time a measure of proliferation. Reduction of 3‐(4,5‐dimethylthiazol‐2‐yl)‐2,5‐diphenyltetrazolium bromide (MTT) to a purple formazan product was employed. Cell metabolic activity was assessed after 24 h, 96 h, and 14 days in culture. At the respective time points, the culture medium was removed; cells were rinsed with phosphate buffered saline (PBS) then incubated in 0.5% MTT/PBS for 40 min at 37°C. The MTT solution was removed and the formazan product was dissolved and eluted using 2‐ethoxyethanol (600 μL per sample). Optical density of eluted solution was determined at 562 nm. Test samples were placed in to fresh wells after incubation in MTT solution and prior to elution of formazan, to ensure that the reactions only included osteoblasts in contact with test surfaces without contribution from cells in the surrounding well. The measured absorbance for the control wells (samples containing no cells) was corrected to take into account the difference in flat (2D) surface area between the samples (1.13 cm^2^) and the wells (2.0 cm^2^).

### Live/Dead Viability Measurement

Osteoblast cells were seeded onto the substrates and cultured for 24 and 96 h. At the respective time points, culture medium was removed and cells incubated in fresh growth medium +0.001% SYTO‐9 (Invitrogen) and 0.0015% (w/v) propidium iodide (Invitrogen, Paisley, U.K.) at 37°C/5% CO_2_ for 15 min. After washing 3× with PBS, osteoblasts were imaged, submerged in PBS using a Zeiss LSM 510 META upright confocal microscope with an Achroplan 10×/0.3 W water dipping objective (working distance = 3.1 mm) using an argon ion laser for SYTO‐9 (λex = 494 nm/λem = 515 nm) and helium–neon laser for propidium iodide (λex = 536 nm/λem = 617 nm)/pinhole = 1 Airy unit. Three fields‐of‐view (840 × 840 μm^2^) were imaged per sample and samples processed for 3D imaging with z‐depths of up to 1,400 μm. Z‐slice intervals were 8.4 and 17.5 μm for Ti VPS surfaces and lattice structures, respectively.

### Cytoskeletal Organization

Osteoblasts were seeded onto the substrates and cultured for 24 and 96 h. Culture medium was removed and cells were fixed in 3.7% (w/v) formaldehyde/PBS for 20 min, washed with PBS and then permeabilized using 0.2% (w/v) Triton X‐100/PBS for 15 min (RTP). After washing 3× with PBS, osteoblasts were incubated for 30 min in the dark (RTP) in PBS containing 0.002% (w/v) phalloidin‐TRITC (to label actin filaments) and 300 nM DAPI (to identify nuclei). Osteoblasts were then washed 3× with PBS and imaged wet, submerged in PBS using a Zeiss LSM 510 META upright confocal microscope using a helium‐neon laser (543 nm) for TRITC excitation (λex = 540–545 nm/λem = 570–573 nm) and a Ti‐Sapphire two‐photon laser (800 nm) for DAPI excitation (λex = 350 nm/λem = 470 nm). Images were captured using Achroplan 10×/0.3 W and Achroplan 40×/0.8 W water dipping objectives with working distances of 3.1 and 3.6 mm, respectively/pinhole = 1 Airy unit. Samples were processed with z‐depths of up to 1,800 μm. The mean z‐slice intervals at 10× magnification were 7.2 and 10 μm for Ti VPS surfaces and lattice structures, respectively and 1.2 μm at 40× magnification for all surfaces.

### Mineralization

Xylenol orange, a non‐toxic calcium‐chelating fluorochrome that labels newly formed calcified material (Wang et al., 2006), was used to observe mineralized nodules at 14 days. Osteoblasts were seeded onto substrates in primary human osteoblast growth medium and after 24 h changed to Osteoblast Differentiation Medium (ECACC/Cell Applications Inc.), changed every 3 days. After 13 days 0.001% (w/v) xylenol orange was added to osteoblasts overnight, removed and washed with PBS × 3. Stained samples were imaged submerged in PBS using a Zeiss LSM 510 META upright confocal microscope with an Achroplan 10×/0.3 W water dipping objective (working distance = 3.1 mm) and a helium‐neon laser for xylenol orange excitation (λex = 440/570 nm/λem = 610 nm).

### Statistical Analysis

Statistical analysis was performed using a two‐tailed Student's *t*‐test. Data were reported as mean ± SEM. *P*‐values less than 0.05 were considered significant.

## Results

The tests performed on the different biomaterial surfaces in this study are summarized in Table Ia.

### Physical Characterization of Surfaces

**Figure 2:**
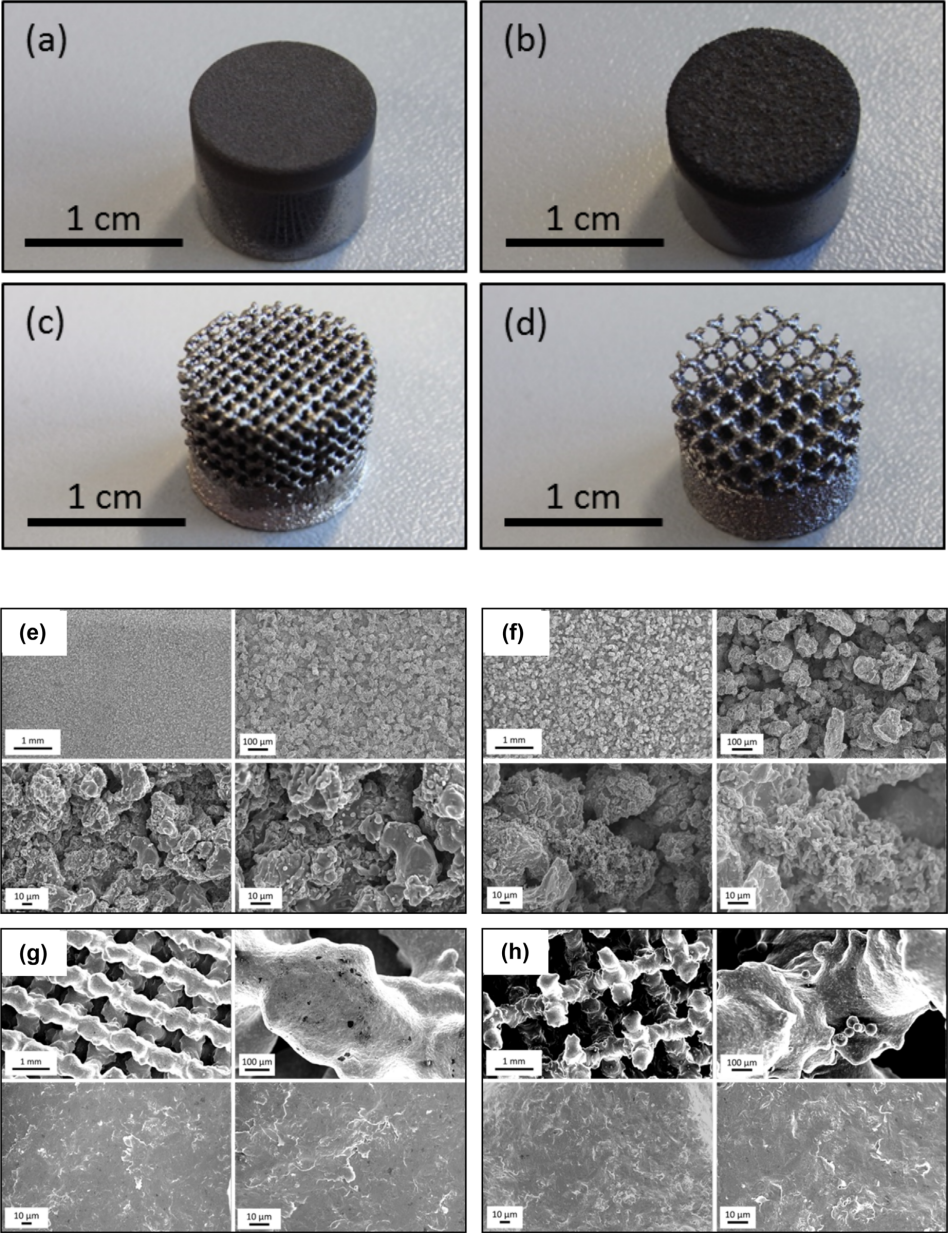
Photographs of the test surfaces investigated in this work: (**a**) Ti Fine, (**b**) Ti Coarse, (**c**) 1.5 mm^3­^ unit cell lattice, and (**d**) 2.5 mm^3^ unit cell lattice. SEM images of (**e**) Ti Fine, (**f**) Ti Coarse, (**g**) 1.5 mm^3^ unit cell lattice, and (**h**) 2.5 mm^3^ unit cell lattice. Images were obtained at magnifications of 20×, 100×, 500×, and 1,000× to compare the topography of the different surfaces.

Test surfaces had noticeably different morphologies at the macro scale due to the differences in preparation method (Fig. 2). The Ti Coarse surface had a much rougher appearance than the Ti Fine surface as expected due to the larger Ti particle size used for plasma spraying. The 2.5 mm^3^ unit cell lattice (Fig. 2d) had a distinctly more open morphology overall than the 1.5 mm^3^ unit cell lattice (Fig. 2c) as a result of the larger unit cell size. The Ti Fine and Ti Coarse VPS surfaces were shown to be homogeneous but with slightly different morphologies by scanning electron microscopy (Fig. 2e and f). Both surfaces exhibited a granular texture with Ti Fine having smaller particles on the surface, which was clearly observed at lower magnifications. At higher magnifications (500× and 1,000×) the differences between the Ti Fine and Ti Coarse surfaces were less pronounced, with both exhibiting a semi porous structure. The coatings were composed of fully melted layers as well as partially melted Ti particles on the surface, resulting in a semi porous structure. The porous lattice substrates exhibited different overall morphologies at low magnifications due to the different unit cell sizes, with the 1.5 mm^3^ unit cell giving a more closed structure than the 2.5 mm^3^ unit cell (Fig. 2g and h). When focussing on individual trusses at higher magnifications (≥100×), there was no difference observed in the surface morphology, irrespective of unit cell size. This was expected as the lattices were manufactured using the same parameters with the only difference being the unit cell size of the lattice structure.

Roughness measurements of the test surfaces were performed to compare the average roughness of the Ti Fine and Ti Coarse VPS coatings with samples manufactured using EBM (Table II). Measurements showed that the Ti Coarse surface had a higher mean surface roughness (Ra) (29.48 μm) than the Ti Fine surface (11.88 μm), which was expected due to the larger Ti powder particle size used for the coarse coating. Measurements on the lattice structures were performed in two directions: horizontally and vertically. Horizontal measurements represent the Z‐direction of the build chamber and the direction in which parts were built, while vertical measurements represent the X‐Y direction of the build chamber. The roughness was slightly higher when measured in the vertical direction; this is due to the layer‐by‐layer nature of the EBM process, which contributes to a rougher surface in the Z‐direction. EBM surface roughness is primarily influenced by the powder size, layer thickness, and orientation. Since it was not possible to measure the surface of the individual trusses, the measurements taken on the solid base were the best approximations available using the stylus technique. The surface roughness of the EBM manufactured surfaces was similar to that of the Ti Coarse coating, with the surfaces having Ra values of 31.33 and 29.48 μm, respectively.

**Table II:** Surface roughness measurements obtained for test surfaces

Surface	Ra (μm)				RzDIN (μm)			
	1	2	3	Mean	1	2	3	Mean
Fine titanium VPS	10.59	12.32	12.73	11.88	49.83	60.27	62.97	57.69
Coarse titanium VPS	33.15	31.23	24.06	29.48	122.41	119.8	107.26	116.49
Lattice structure (vertical)	38.25	25.67	30.05	31.33	141.46	115.71	120.39	125.85
Lattice structure (horizontal)	24.47	32.93	29.06	28.83	105.37	125.35	82.28	104.34

### Osteoblast Cell Metabolic Activity

**Figure 3:**
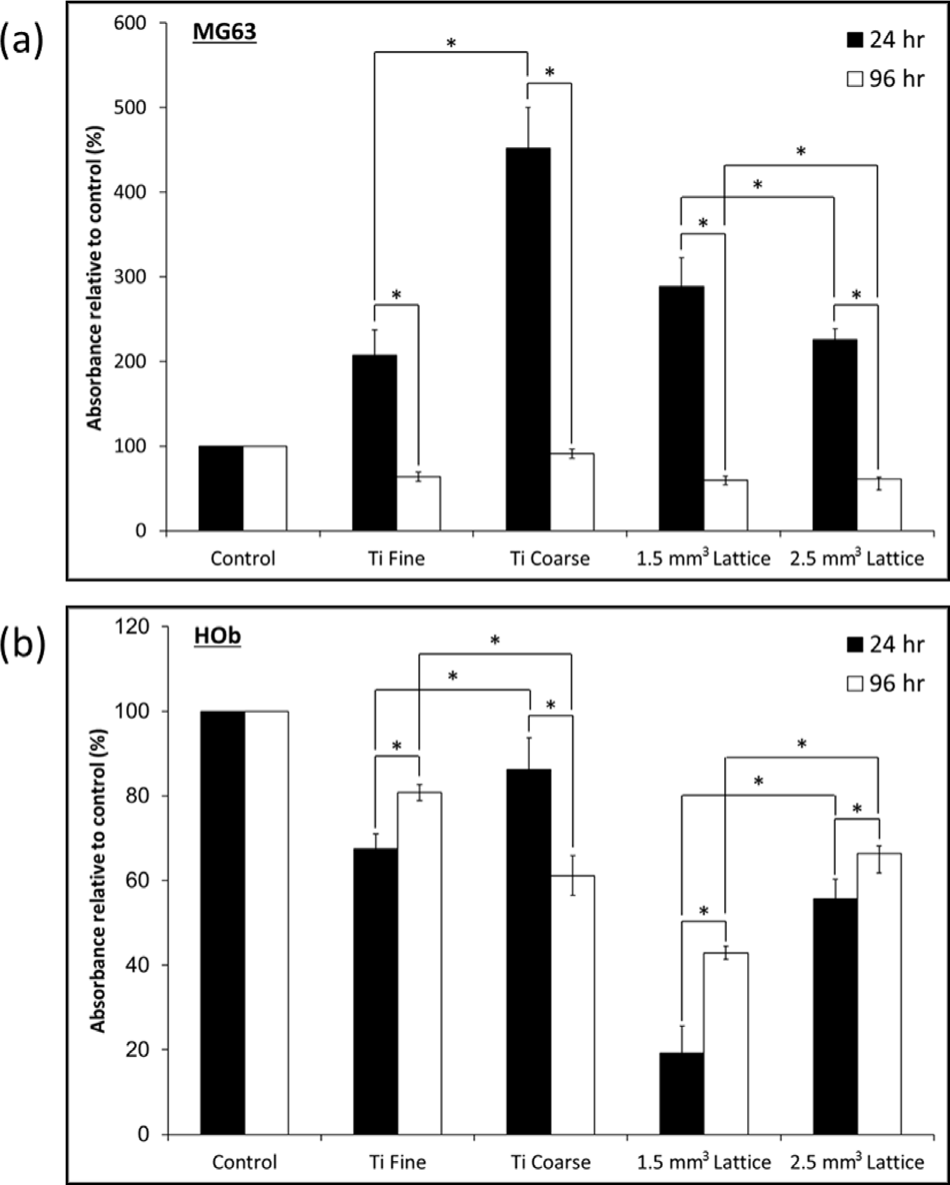
Metabolic activity of (**a**) MG63 cell line and (**b**) HOb cells on test surfaces assessed using MTT assay after 24 and 96 h culture. Values presented are the mean average ± SE (*n* = 3).

The metabolic activity of the MG63 cell line and primary human osteoblast cells on test surfaces was determined after 24 and 96 h in culture using an MTT assay, with control experiments performed on tissue culture plastic. Test surface data was expressed as a percentage of corresponding control values, where were the control surfaces were processed in parallel but without cells. For MG63 cells, metabolic activity decreased between 24 and 96 h on all surfaces (Fig. 3a), and thus a decrease in cell number arose. Higher MG63 metabolic activity was observed on all test surfaces compared to control at 24 h. The increase in metabolic activity observed on the test surfaces between the two time points was not as great as the increase in metabolic activity on the control surface, indicating difference in proliferation on the surfaces. Primary human osteoblasts showed a significant increase in metabolic activity, and hence proliferation, between 24 and 96 h on all surfaces, except on the Ti Coarse surface (Fig. 3b). For the VPS surfaces, osteoblasts on the Ti Fine material had significantly lower metabolic activity than on the Ti Coarse material at 24 h. However, at 96 h the metabolic activity on Ti Fine was significantly higher than that on Ti Coarse relative to control. This was of note, indicating an increase in proliferation. There was a significant increase in the metabolic activity of primary human osteoblasts on the porous lattice substrates between 24 and 96 h for both unit cell sizes. Primary osteoblasts also had a significantly higher metabolic activity on the 2.5 mm^3^ lattice than on the 1.5 mm^3^ lattice at both time points.

### Osteoblast Cell Live/Dead Measurement

**Figure 4:**
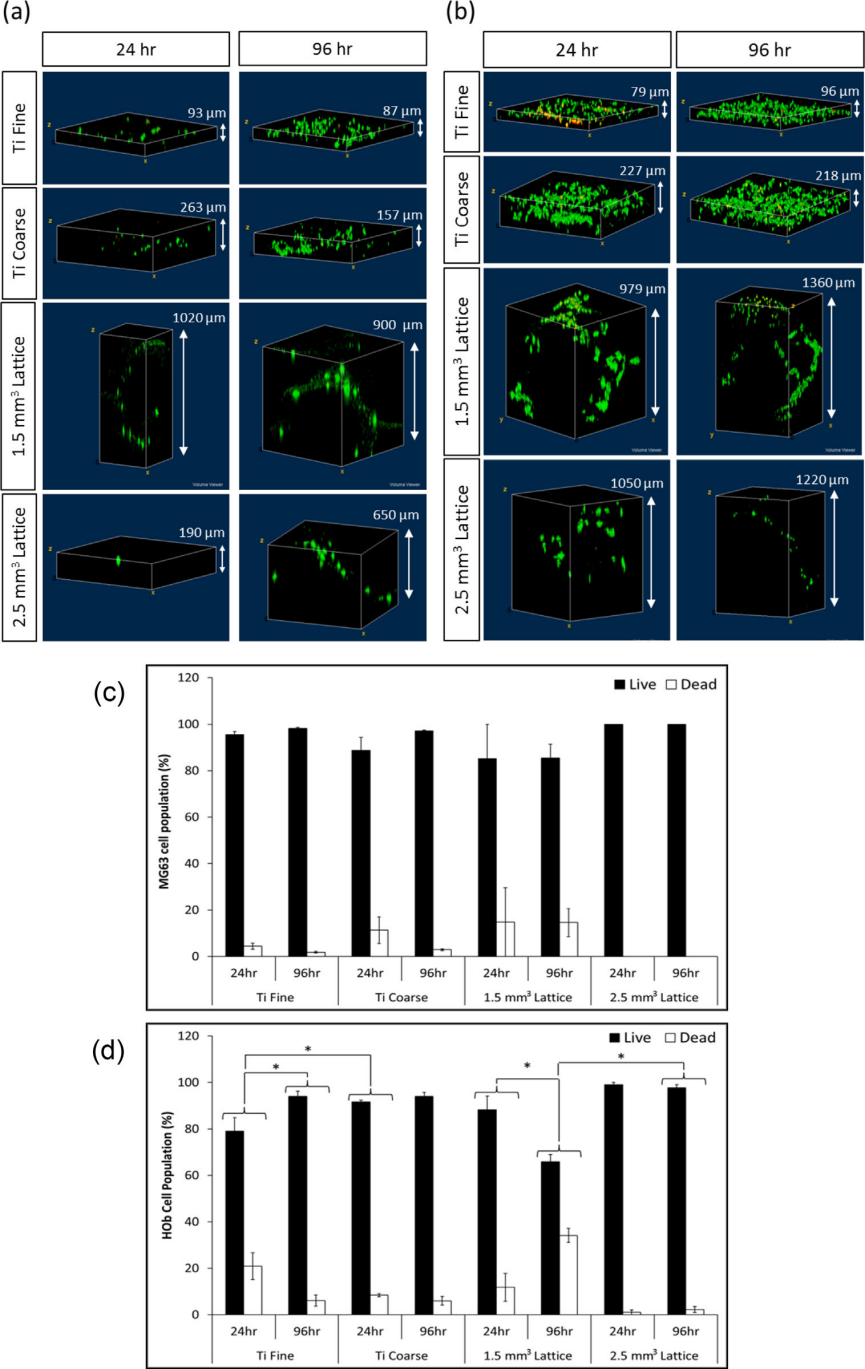
Representative confocal micrographs of (**a**) MG63 osteosarcoma cells and (**b**) HOb cells labelled with SYTO9 (live cells, green) and propidium iodide (dead cells, red) cultured on the different surfaces at 24 and 96 h. Live/dead analysis of (**c**) MG63 cell line and (**d**) HOb cells on different test surfaces. The proportion of live/dead cells was calculated from the mean average (±SE) cell number counted across three fields‐of‐view (840 × 840 μm^2^) per sample (*n* = 3). *Statistically significant difference (*P* < 0.05) when compared using Student's *t*‐test.

No significant difference was observed in the percentage of live MG63 cells for all surfaces at 24 and 96 h, with >80% live cells (Fig. 4a and c). Cells were distributed across the surfaces, with no clusters of dead cells observed. Cells were dispersed throughout the porous lattice structures and as such it was difficult to obtain fields of view containing many cells due to the low cell density, particularly at 24 h on the more open 2.5 mm^3^ lattices. There was a significantly lower proportion of live primary human osteoblast on the Ti Fine material at 24 h (Fig. 4b and d; 79%) compared to 96 h (94%). A significantly lower proportion of live primary human osteoblasts were observed at 24 h on the Ti Fine surface compared to Ti Coarse at the same time point (92%). This difference was not observed at 96 h; with 94% live osteoblasts observed on both Ti Fine and Ti Coarse surfaces. Osteoblasts were evenly distributed across both the Ti Fine and Ti Coarse substrates, with dead cells dispersed across the surfaces and no clustering observed (Fig. 4d). For the lattice structures, there was no significant difference between the unit cell sizes in the proportion of live cells at 24 h (Fig. 4b). However, at 96 h the 1.5 mm^3^ lattice had a significantly lower proportion of live cells than the 2.5 mm^3^ lattice. The dead cells on the 1.5 mm^3^ lattice (96 h) were concentrated at the end of the trusses on the top of the lattice (Fig. 4d), with very few dead cells observed on the trusses deeper into the lattice structure.

### Osteoblast Cell Morphology and Cytoskeletal Organization

**Figure 5:**
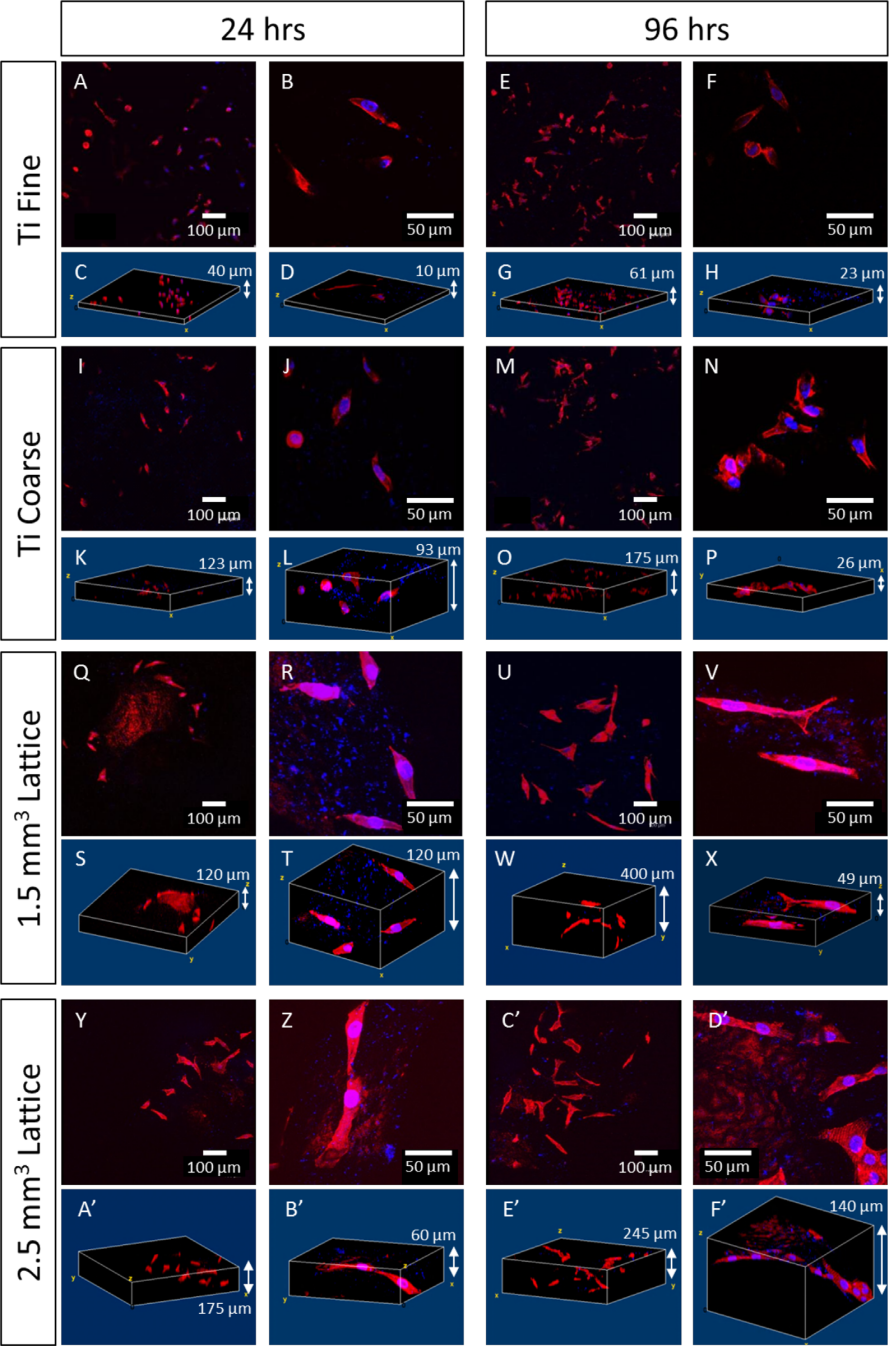
Confocal micrographs of MG63 osteosarcoma cells labelled for actin filaments (phalloidin‐TRITC, red) and nuclei (DAPI, blue), respectively, cultured on test surfaces for 24 and 96 h. Corresponding 3D micrographs show the distribution and phenotype of cells on the different surface topographies.

**Figure 6:**
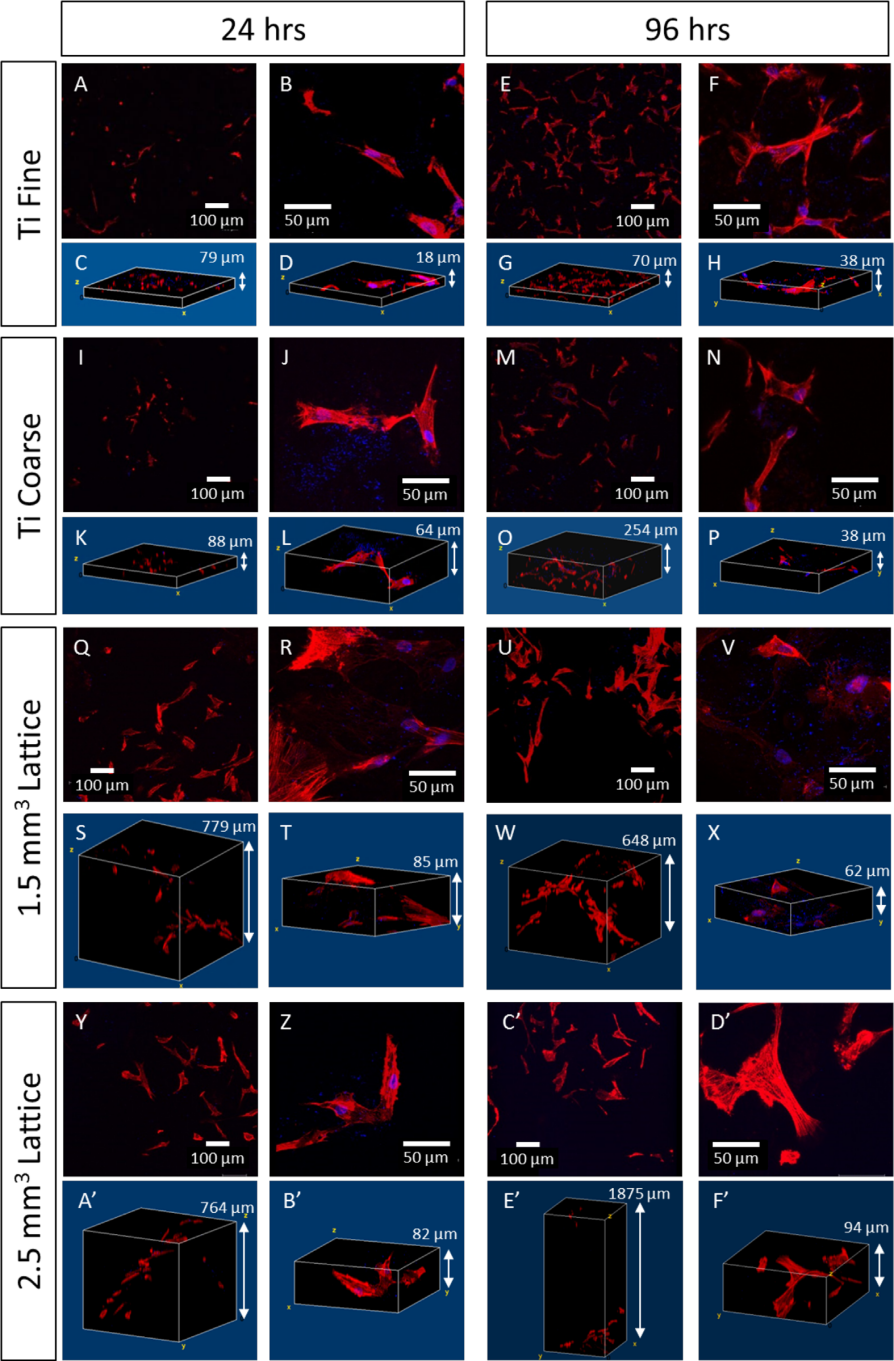
Confocal microscopy images of primary human osteoblast cells (HOb) labelled for actin filaments (phalloidin‐TRITC, red) and nuclei (DAPI, blue), respectively, cultured on test surfaces for 24 and 96 h. Corresponding 3D images are presented to show the distribution and shape of cells across the different surface topographies tested.

To evaluate the shape of cells on the test surfaces, actin filaments and cell nuclei were labelled with phalloidin‐TRITC and DAPI, respectively, after 24 and 96 h in culture, by z‐stack confocal microscopy (Fig. 5 [MG63 cells] and Fig. 6 [primary osteoblasts]) at low (10×) and higher (40×) magnification. On Ti Fine, MG63 cells were observed to have a rounded morphology by 24 h; however, some actin filament organization was observed (Fig. 5a–h). By 96 h, a greater number of cells had adopted a fibroblastic appearance and were spread across the surface with actin filaments observed and formation of focal adhesions, indicating adherence to the substrate. A similar response was observed for MG63 cells cultured on the Ti Coarse substrate (Fig. 5i–p), with cells initially displaying a rounded morphology at 24 h, but becoming more fibroblastic by 96 h. In general, cells remained more rounded on the Ti Coarse substrate than Ti Fine, even after 96 h.

Z‐stack imaging demonstrated that the cells were rounded, but by 96 h cells had re‐modeled as actin filaments were formed, with cells displaying filopodia to attach to the substrate surface and lengthen overall and over a greater surface area. Z‐stacks revealed cells present to a greater on the Ti Coarse surface. On the porous lattice surfaces, MG63 cells exhibited an elongated, fibroblastic structure after 24 h in culture, with filament organization clearly observed (Fig. 5q–t and y–b′). The morphology of the lattice trusses (Fig. 2) would be expected to be less constraining on the cells than the VPS Ti surfaces, and therefore more permissive for cells to elongate across the surface. After 96 h, there was an increase in cell number; however, little difference in cell morphology was observed compared to 24 h (Fig. 5u–x and c′–f′). Cells remained elongated with a fibroblastic shape, actin filament structures and focal adhesion formation was observed. Cells were clearly positioned along the lattice structures, both in overall distribution (Fig. 5e′), which could be observed at an individual cell level (Fig. 5f′). The response was similar on all lattice structures, irrespective of unit cell size.

Primary human osteoblast cells showed an elongated appearance after 24 h in culture on Ti Fine and Ti Coarse surfaces (Fig. 6a–d and i–l), with few rounded cells observed. At higher magnification, filament organization was clearly seen with filopodia extending from the cells across the surfaces and focal adhesions being formed (Fig. 6b–j). After 96 h in culture, there was an increase in cell number across both the Ti Fine and Ti Coarse surfaces. Osteoblast cells formed clear focal adhesions on both fine and coarse surfaces with filament organization observed and a less fibroblastic, more polygonal shape, characteristic of osteoblasts. The distribution of cells across the rougher Ti Coarse surface was demonstrated in z‐stack images, with cells observed over a greater z‐depth on Ti Coarse (284 μm) than Ti Fine surfaces (70 μm).

Primary human osteoblast cells had a fibroblastic shape after 24 h in culture on the porous lattice substrates (Fig. 6q–t and y–b′), with actin filaments observed at higher magnification. After 96 h, cells remained fibroblastic, with some becoming polygonal. Filament organization was clearly observed with physical position closely following the surface shape of trusses and forming focal adhesions (Fig. 6f′). The distribution of cells across lattice structures was observed by z‐stack, with cells closely following the lattice structure on both the 1.5 and 2.5 mm^3^ unit lattices (Fig. 6).

### Mineralization

**Figure 7:**
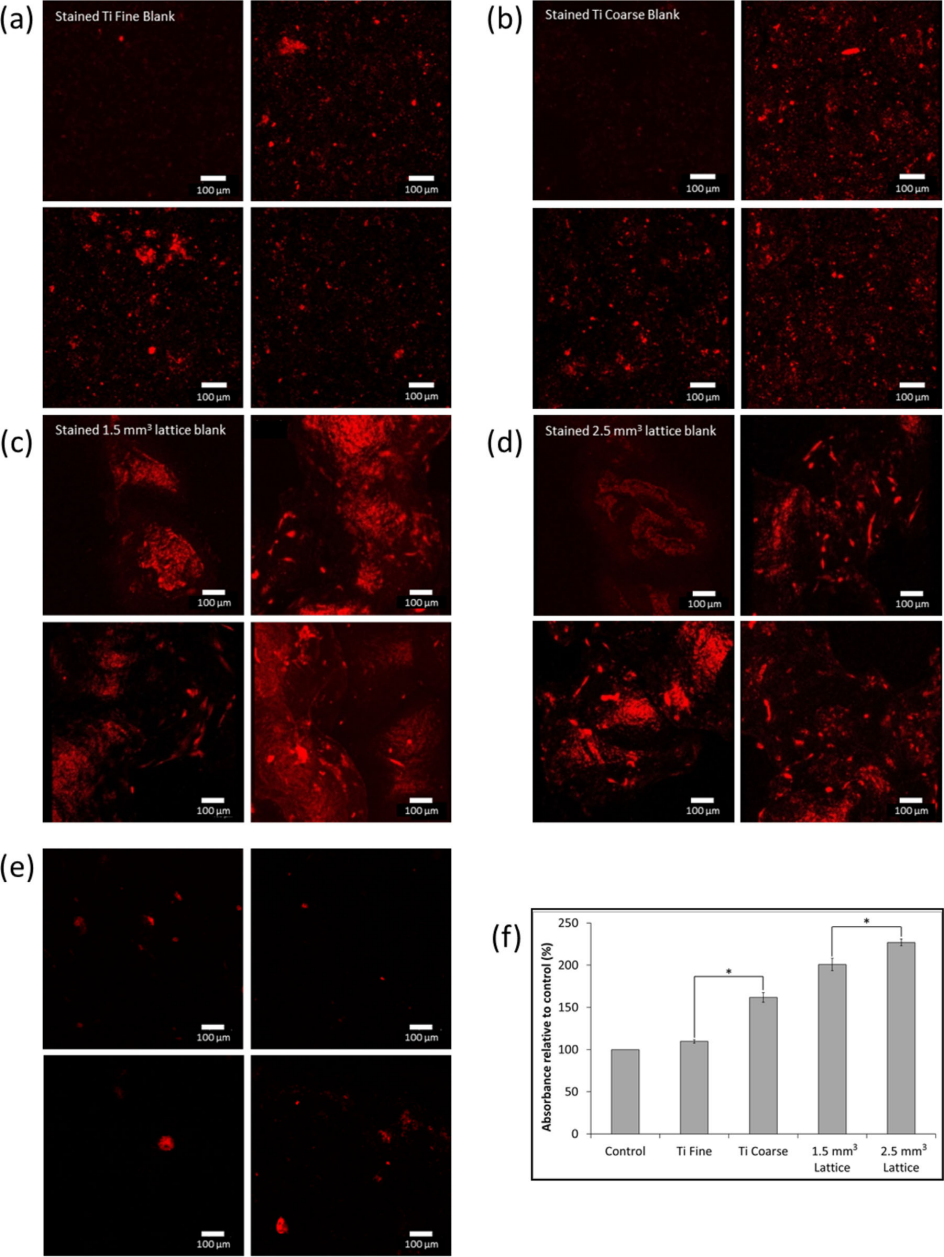
(**a–e**) Confocal micrographs of xylenol orange stained mineralized deposits of HOb cultured for 14 days on (**a**) Ti Fine, (**b**) Ti Coarse, (**c**) 1.5 mm^3^ unit cell lattice, (**d**) 2.5 mm^3^ unit cell lattice, and (**e**) tissue culture plastic (control). For test surfaces (**a–d**), images of xylenol orange stained blank samples (without cells) are included for comparison. (**f**) Metabolic activity of HOb cells on test surfaces after 14 days in culture. Values are mean ± SE (*n* = 3). *Statistically significant difference (*P* < 0.05) by Student's *t*‐test.

The mineralization of primary human osteoblast cells was measured after 14 days in culture using xylenol orange staining and confocal microscopy (Fig. 7a–e). Cell metabolic activity was also assessed at the same time point by MTT assay (Fig. 7f). To distinguish between background fluorescence and cell response, blank samples (no cells) of each test surface were stained with xylenol orange and imaged by confocal microscopy. Figure 7a–d shows representative images of xylenol orange stained osteoblasts on test surfaces. Positively stained nodules were observed on all test surfaces, indicative that osteoblast mineralization had occurred. A greater number of positively stained nodules were observed on the test surfaces versus control (tissue culture plastic) (Fig. 7e), which correlated with osteoblast metabolic activity (Fig. 7f). An increase in positively stained nodules was observed on Ti Course and Ti Fine surfaces, compared to control. The size of the nodules/mineralized areas appeared to be greater on Ti Fine than on Ti Coarse. For primary human osteoblast cells cultured on the lattice substrates, stained nodules were clearly observed above the fluorescent background, indicating that mineralization had occurred. There was little difference between the nodules observed on the lattice structures, irrespective of the unit cell size. Some background fluorescence was observed for the porous lattice structures, due laser reflection.

## Discussion

We report on the development of a human‐based 3D in vitro approach for testing a range of candidate orthopaedic materials. The approach combined information from a bone‐like osteosarcoma MG63 cell line and normal primary human adult osteoblast cells. Confocal laser scanning microscopy was used to study cell distribution across rough and porous surfaces to investigate cellular response to the different surface topographies. All surfaces consisted of titanium in a commercially pure form (plasma sprayed coating) and/or the alloy Ti6Al4V (reported to be biocompatible and bioinert Hench and Polak, 2002). The development of an additive manufacturing process enabled the formation of complex surface geometries that cannot be manufactured by traditional means. It is important that surfaces prepared by such new technologies, even if using traditional materials, are assessed for biocompatibility and evaluate cell response.

Medical device manufacturers are increasingly required to perform extensive assessments of new biomaterials before regulatory approval is achieved. In vitro testing enables the rapid assessment of prototype surfaces, providing information for informed decisions to be made on which to take forward before in vivo and/or clinical investigation. Here, confocal laser scanning microscopy enabled the evaluation of human osteoblast cells across a range of complex surface shapes (Georgakoudi et al., 2008), and provides an initial indication of how osteoblasts may behave in vivo. This has a major advantage of reducing the time and cost for surface selection during medical device development. Separately, there is an increasing emphasis of alternative methods to animal models for materials screening, in part driven by the “3Rs” (Replacement, Refinement, and Reduction of animals in scientific research), first introduced by Russell and Burch (1959), and supported by an increasing number of countries worldwide (e.g., in the UK by the National Centre for the Replacement, Refinement, and Reduction of Animals in Research (NC3Rs).

The in vitro assessment of materials has an advantage of having relatively well‐controlled variables, which enable the investigation of parameters otherwise difficult to study in vivo (Schmidt et al., 2001). The use of human cells is another advantage—here an MG63 osteoblast‐like cell line (Chang et al., 2014; Gittens et al., 2011; Madhankumar et al., 2014; Tan et al., 2011; Yazici et al., 2013; Yeung et al., 2013) and primary human osteoblast cells (Balani et al., 2007; Gough et al., 2004; Jones et al., 2007; Rice et al., 2003) were used. Although the MG63 cell line is immortalized (Schmidt et al., 2001), such cells are suitable for establishing initial experimental conditions. Normal primary human osteoblasts were used thereafter to study markers including differentiation (with the caveat that all human primary cells are inherently variable in the genetic and phenotypic response) to identify whether similar responses were observed across the orthopaedic surfaces. The methods developed in this work combine some well‐established in vitro techniques alongside some novel 3D imaging approaches for evaluating biomaterial suitability.

In material terms the test surfaces were very similar, but the topographies differed greatly (Fig. 2) and elicited differences in osteoblast response. An increase in metabolic activity of both cell types was observed over time on Ti VPS surfaces, with metabolic activity found to be higher on Ti Coarse versus Ti Fine surfaces after 24 and 96 h culture (Fig. 3), consistent with an increase in cell number. This may have been due to an increased surface area and surface roughness of the coarse coated surface. Surface roughening has been shown previously to have a stimulatory effect on cell response to titanium substrates (Gittens et al., 2011). These findings correlated with live/dead data, where Ti Coarse was found to support a higher proportion of live cells at both time points studied (Fig. 4). There was little difference in overall in the primary human osteoblast number between 24 and 96 h time points on the Ti Fine surface (Fig. 4); however, an increase in the proportion of live cells was observed, which correlated with increased viability. The differences in osteoblast survival were not observed with the MG63 cell line, highlighting the greater sensitivity of primary human cells to changes in surface environment. It was also noted that the Ti Fine surface appeared to have much greater hydrophobicity than the Ti Coarse surface. Some sample areas did not appear to be fully wetted, with osteoblasts not adhering or proliferating in those areas. This would correlate with overall cell number on the surface contributing to the lower metabolic activity measured.

Differences in osteoblast phenotype were observed between surfaces, particularly for MG63 cells, where a much more rounded shape on the Ti Coarse arose (Fig. 5). This may have been due to deep troughs on the rough surface, physically constraining cells to form a rounded shape until they were able to elongate and spread further across the surface. However, this was not seen for primary human osteoblasts. In contrast, focal adhesion formation and a characteristic polygonal osteoblastic phenotype was observed, even after 24 h culture on both surfaces (Fig. 6). Cell attachment to surfaces is vital for cell survival and essential for subsequent mineralization and bone formation to occur (Grigoriou et al., 2005). Xylenol orange staining indicated a qualitatively greater degree of primary human osteoblast mineralization on the Ti Coarse surface (Fig. 7). This was supported by metabolic activity measurements, which showed a greater activity for Ti Coarse at the 14‐day time point (Fig. 7f). The results suggest that of the vacuum plasma sprayed titanium surfaces assessed the greater roughness of the Ti Coarse surface correlated with higher levels of cell metabolic activity and mineralization and would lead us to conclude was a more favorable material for orthopaedic applications.

MG63 cells showed greater metabolic activity on the 1.5 mm^3^ porous lattices at 24 h; however, by 96 h there was no significant difference between the lattice surfaces (Fig. 3). This did not correlate with live/dead data, which showed a lower proportion of live cells at both time points on the 1.5 mm^3^ lattice (Fig. 4). It is important to note that the live/dead data obtained was semi‐quantitative and the absolute number of MG63 cells imaged was low across the samples (<20/sample area), due to the depth and solid nature of the lattice trusses and the cell seeding density used. Primary human osteoblast assessment showed an opposite trend with greater cell metabolic activity observed for the 2.5 mm^3^ lattice at both time points. There was a significant decrease in the proportion of live primary human osteoblast on the 1.5 mm^3^ lattice between time points, although there was no overall change in the number of cells imaged. A concurrent increase in cell viability was observed on this surface. Confocal microscopy of osteoblasts labelled with SYTO9/propidium iodide and phalloidin‐TRITC for live/dead and actin analysis, revealed that primary human osteoblasts were distributed along the trusses for both lattice dimensions (Figs. 4 and 6). Cell organization along the trusses was clearly observed, with images obtained of z‐depths up to 1,875 μm for some test samples. Xylenol orange staining indicated a greater degree of osteoblast mineralization on the 2.5 mm^3^ lattice. This was supported by a higher cell metabolic activity observed for the 2.5 mm^3^ lattice at the same 14‐day time point (Fig. 7f). A difference in osteoblast response was not anticipated between the lattice surfaces as the only variation was in unit cell size. The lattices were fabricated using the same manufacturing technique, which produced analogous truss surface morphologies on the micrometre scale as evidenced by SEM imaging (Fig. 2). However, significant differences in viability of primary human osteoblast cells was observed by live/dead analysis, with significantly lower proportions of live cells observed on the 1.5 mm^3^ lattices. Dead cells appeared to be concentrated on the upper end of the trusses (Fig. 4b). The results therefore indicate that the 2.5 mm^3^ lattice was a more favorable substrate for osteoblast cells, with greater cell viability for both types and a higher degree of mineralization of primary human osteoblast cultures observed.

## Conclusions

In summary, a series of preclinical/pre‐in vivo methods were developed to rapidly screen potential biomaterials surfaces for orthopaedic applications. In vitro techniques were combined with 3D CLSM to evaluate human osteoblast responses to titanium surfaces created using conventional plasma spraying and emerging additive manufacture technologies, notably EBM. Primary human osteoblast cells were found to elicit greater differences between test surfaces than the MG63 osteoblast‐like cell line, particularly in terms of cell survival. Surface morphology was found to influence cell response with higher metabolic activity and mineralization observed on the rougher plasma spray coating (Ti Coarse) than the smoother (Ti Fine) surfaces. Differences in the survival and metabolic metabolic activity of primary human osteoblast on lattice structures with different unit cell sizes were also found, despite analogous surface morphology at the cellular level. 3D z‐stack confocal microscopy was particularly valuable for assessing cellular organization within complex surface geometries, with cell organization identified as adhering along the strut geometries. This, in conjunction with mineralized nodule formation throughout the lattice structures was observed, and indicative of early markers of bone in‐growth on such materials. The combination of in vitro methods was concluded as being valuable as a rapid, accurate, and cost‐effective approach for assessing potential materials being developed for orthopaedic applications.

We acknowledge funding from JRI Orthopaedics Ltd., Innovate U.K. (formerly the Technology Strategy Board), EPSRC, BBSRC, and the Department of Health through the Knowledge Transfer Partnership Scheme, U.K. (KTP008411). Imaging work was performed at the Kroto Research Institute Confocal Imaging Facility, University of Sheffield, U.K., with the assistance of Dr. Nicola Green.
